# Metabolomic profile of patients on levothyroxine treatment for hypothyroidism

**DOI:** 10.1530/ETJ-23-0062

**Published:** 2023-07-27

**Authors:** Hicham Benabdelkamel, Malak A Jaber, Lina A Dahabiyeh, Afshan Masood, Reem H Almalki, Mohthash Musambil, Anas M Abdel Rahman, Assim A Alfadda

**Affiliations:** 1 Proteomics Resource Unit, Obesity Research Center, College of Medicine, King Saud University, Riyadh, Saudi Arabia; 2 Pharmaceutical Medicinal Chemistry & Pharmacognosy, Faculty of Pharmacy and Medical Sciences, University of Petra, Amman, Jordan; 3 Division of Pharmaceutical Sciences, School of Pharmacy, The University of Jordan, Amman, Jordan; 4 Metabolomics Section, Department of Clinical Genomics, Center for Genome Medicine, King Faisal Specialist Hospital and Research Center, Riyadh, Saudi Arabia; 5 Department of Botany and Microbiology, College of Science, King Saud University, Riyadh, Saudi Arabia; 6 Department of Biochemistry and Molecular Medicine, College of Medicine, Alfaisal University, Riyadh, Saudi Arabia; 7 Department of Medicine, College of Medicine and King Saud Medical City, King Saud University, Riyadh, Saudi Arabia

**Keywords:** thyroid hormone, hypothyroidism, LC-MS based metabolomics, levothyroxine, lipid disturbance, anabolic effect, bile acid, metabolomics biomarker

## Abstract

**Background:**

Hypothyroidism is clinically characterized by a decrease in levels of the circulating thyroid hormones namely thyroxine and triiodothyronine. The main treatment for hypothyroidism is thyroid hormone replacement using levothyroxine to normalize serum thyroid hormone levels.

**Objectives:**

In this study, we explored the metabolic changes in the plasma of patients with hypothyroidism after reaching a euthyroid state with levothyroxine treatment.

**Methods:**

Plasma samples from 18 patients diagnosed as overt hypothyroidism were collected before and after levothyroxine treatment upon reaching a euthyroid state and were analyzed by high-resolution mass spectrometry-based metabolomics. Multivariate and univariate analyses evaluated data to highlight potential metabolic biomarkers.

**Results:**

Liquid chromatography-mass spectrometry-based metabolomics revealed a significant decrease in the levels of ceramide, phosphatidylcholine, triglycerides, acylcarnitine, and peptides after levothyroxine treatment; this could indicate a change in the fatty acid transportation system and an enhanced β-oxidation, compared with a hypothyroid state. At the same time, the decrease in the peptides suggested a shift in protein synthesis. In addition, there was a considerable rise in glycocholic acid following therapy, suggesting the involvement of thyroid hormones in stimulating bile acid production and secretion.

**Conclusions:**

A metabolomic analysis of patients with hypothyroidism revealed significant changes in several metabolites and lipids after treatment. This study showed the value of the metabolomics technique in providing a complementary understanding of the pathophysiology of hypothyroidism and as a crucial tool for examining the molecular impact of levothyroxine treatment on hypothyroidism. It was an important tool for investigating the therapeutic effects of levothyroxine on hypothyroidism at the molecular level.

## Introduction

The thyroid hormone affects almost every organ system in the body, including the cardiovascular, central nervous, skeletal, and gastrointestinal systems and metabolism. Tetraiodothyronine or thyroxine (T_4_) and triiodothyronine (T_3_) are the main hormones released by the thyroid gland (1). The hypothalamus and anterior pituitary gland produce and release several hormones that work synchronously to maintain the proper feedback mechanisms and homeostasis of T_4_ and T_3_ in the bloodstream (2).

Numerous *in vitro* and *in vivo* previously conducted studies on animals and humans have examined the effect of thyroid hormones on metabolic balance (3). The research findings prompted the development of different diagnostic tests and therapeutic agents now implemented in the clinical setting. To assess thyroid gland function, several blood tests can be used to measure the levels of thyroid hormones and other related hormones. These tests include those for thyroid-stimulating hormone (TSH), total T_4_ and T_3_, and free T_4_ (FT_4_) and T_3_. The other tests for thyroid antibodies and thyroglobulin identify autoimmune thyroid conditions and thyroiditis, respectively (4).

Hypothyroidism, or an underactive thyroid, is a medical condition characterized by a decrease in the thyroid hormones released into the bloodstream (5, 6). It is clinically distinguished by an indirect but broad range of signs, depending on the severity of the hormone deficiency that reflects a hypometabolic state. This metabolic condition is marked by decreased basal metabolic rate, heat production, and increased resting energy expenditure and is associated with increased cholesterol levels, decreased lipolysis, and decreased gluconeogenesis (7).

Hypothyroidism is divided into primary, secondary, and tertiary stages. In primary hypothyroidism, diminished thyroid gland production of hormones causes a compensatory increase in TSH. On the other hand, secondary hypothyroidism is characterized by decreased TSH and T_3_/T_4_ levels and is usually caused by pituitary disorders (8). Tertiary hypothyroidism is caused by hypothalamic disorders and results in decreased levels of thyrotropin-releasing hormone released from the hypothalamus, TSH, and T_3_/T_4_ (8).

Thyroid hormone replacement is currently the primary treatment method for hypothyroidism (6). According to patient feedback, levothyroxine (LT_4_) is a synthetic replacement for thyroid tests. The recommended dose for adults is 1.6 µg/kg, adjusted every 8 weeks, depending on the response and thyroid test results. Although patients may have partial symptom alleviation with medicine intake, they typically experience over replacement, which makes them more vulnerable to osteoporosis, liver damage, and cardiovascular diseases. Therefore, novel approaches to managing hypothyroidism are urgently needed.

Metabolomics is a well-known and widely utilized approach to understanding perturbation in an organ system under stress. This approach can provide novel insights into the end product of various biochemical processes within the body, which could reveal the physiological status of that system and identify new therapeutic targets (9, 10, 11, 12, 13, 14, 15). In hypothyroidism, metabolomics has been applied to understand the molecular mechanism in the urine of rats (16), detect metabolic changes associated with the transition from hypothyroidism to euthyroidism using NMR-based metabolomics (17), identify biomarkers to distinguish between hypothyroid and euthyroid individuals (9), and evaluate the effect of herbs with hot properties on hypothyroidism rats (18). The current study used a metabolomic technique based on liquid chromatography-mass spectrometry (LC-MS) to evaluate the metabolic changes connected with hypothyroidism therapy.

## Materials and methods

### Ethics approval, patient recruitment, and treatment

The study was approved by the King Khalid University Hospital (KKUH), College of Medicine, Riyadh, Saudi Arabia (registration no. E-10-172). The recruited patients, referred to the KKUH Obesity Research Center, were asked to sign a written informed consent form before enrolment. Eighteen overweight patients (10 women and 8 men) with BMI ranging from 25 to 27 kg/m^2^ who were diagnosed as overt hypothyroidism were included in this study. TSH values > 10 mIU/L and FT_4_ levels < 12 pmol/L were considered to indicate overt hypothyroidism. All patients were in good health and had no other pathological disorders, such as type 2 diabetes, hypertension, and inflammatory or autoimmune diseases.

The patients were treated with an appropriate dose of LT_4_. Samples for the hypothyroid group were taken before initiation of treatment, and the post-treatment samples were collected after the TSH levels were normalized after giving the recommended dose. The TSH levels normalized in 15 patients after 6 weeks of therapy, while 2 patients took 8 weeks and 1 patient took 10 weeks to reach euthyroid levels.

### Biochemical analysis

Standard clinical tests were performed at the first visit to the hospital after diagnosis and repeated when a euthyroid state was achieved. The following parameters were evaluated using blood samples that were obtained after overnight fasting: FT_4_, TSH, fasting blood glucose, urea, creatinine, sodium, potassium, aspartate transaminase, alanine transaminase, alkaline phosphatase, total cholesterol, low-density lipoprotein cholesterol (LDL), triglyceride (TG), and high-density lipoprotein cholesterol. An integrated clinical chemistry autoanalyzer (Dimension® Xpand Plus; Siemens Healthcare Diagnostics) was used to determine all parameters for biochemical and hormone studies listed in Table 1.

**Table 1:** Biochemical parameters of the hypothyroid patients at baseline and after levothyroxine therapy.

Parameters	Hypothyroid	Euthyroid	*P-*value
Patient number	18		-
Age (years)	39 ± 11.7		-
Female/male	10/8		-
FT_4_ (pmol/L)	7.9 ± 5.8	17.9 ± 3.5	<0.0001^a^
TSH (mIU/L) median (IQR)	23.79 (36.39)	1.20 (1.08)	<0.0001^a^
Fasting blood glucose (mmol/L)	5.2 ± 0.5	5.1 ± 0.3	0.067
Urea (mmol/L)	4.4 ± 0.2	4.8 ± 0.8	0.318
Creatinine (umol/L)	74.5 ± 11.1	75.7 ± 20.4	0.450
Sodium (mmol/L)	140.1 ± 1.7	139.5 ± 0.9	0.79
Potassium (mmol/L)	4.6 ± 0.2	4.6 ± 0.1	1.20
Aspartate transaminase (IU/L)	35.3 ± 7.1	36.8 ± 7.5	0.450
Alanine transaminase (IU/L)	18.8 ± 6.2	17.9 ± 3.5	0.21
Alkaline phosphatase (IU/L)	95.8 ± 19.9	97.2 ± 21.1	0.86
Total cholesterol (mmol/L)	4.5 ± 0.4	4.9 ± 0.8	0.66
Low-density lipoprotein cholesterol (mmol/L)	1.4 ± 0.3	1.2 ± 0.2	0.25
Triglyceride (mmol/L)	1.5 ± 0.2	1.4 ± 0.4	0.35
High-density lipoprotein cholesterol (mmol/L)	2.3 ± 0.7	2.9 ± 0.5	0.32

Values are expressed as means ± s.d. median (IQR) and ^a^indicate significant *P*-value.

FT_4_, free thyroxine; TSH, thyroid-stimulating hormone.

### Sample preparation and liquid chromatography-mass spectrometry analysis

The blood samples were placed in EDTA-coated tubes (BD Vacutainer, USA) and centrifuged at 3000 ***g*** for 15 min; after that, plasma samples were kept for analysis at −80°C (19). For plasma metabolite extraction, proteins were precipitated using a mixture of 50% acetonitrile in methanol. The mixtures were processed in a thermomixer (Eppendorf, Germany) at 600 ***g*** and 4°C for 1 h, followed by centrifugation at 16,000 ***g*** for 10 min at 4°C. The supernatant was transferred into a 1.5 mL tube (Eppendorf) and then evaporated completely in a chamber (SpeedVac; Christ, Germany). The dried samples were suspended using 50% mobile phase A:50% mobile phase B.

Metabolic profiling was performed using a Waters Acquity UPLC system coupled with a Xevo G2-S QTOF mass spectrometer. Metabolites were first separated in a column (100 × 2.1 mm, 2.5 μm) (XSelect; Waters Ltd., Elstree, UK) using ACQUITY UPLC and a binary mobile phase that comprised 0.1% formic acid in dH2O as solvent A and 0.1% formic acid in 50% acetonitrile: methanol as solvent B running at a flow rate of 300 µL/min. The gradient elution was as follows: 0–16 min 95–5% mobile phase A, 16–19 min 5% mobile phase A, 19–20 min 5% to 95% mobile phase A, and 20–22 min 95% mobile phase A. The MS data for the separated metabolites were acquired using positive and negative ionization under the following conditions: source temperature, 150°C; desolvation temperature, 500°C (ESI+) or 140 (ESI−); capillary voltage, 3.20 kV (ESI+) or 3 kV (ESI−); cone voltage, 40 V; desolvation gas flow, 800.0 L/h; and cone gas flow 50 L/h. The collision energy values of the low and high functions were set at off and 10–50 V, respectively, in MSE mode. Quality control samples were prepared by pooling aliquots from all samples and were injected every 10 samples to check the reproducibility of the LC-MS system. All data were collected with Masslynx™ V4.1 workstation (Waters Inc., Milford, MA, USA) in continuum mode.

### Data processing and statistical analysis

Peak picking and alignment of the detected ion (m/z, Rt) were processed using Progenesis QI v.3.0 software (Waters Technologies). Thereafter, the processed data were statistically evaluated using several approaches. After data treatment, including log-transformation, mean centering, and Pareto scaling, MetaboAnalyst v5.0 (McGill University, Montreal, QC, Canada) was used to highlight the metabolic changes associated with LT_4_ treatment (18). In addition, in order to maximize the covariance between the measured data (peak intensities) and the response (class assignment) within the groups, the supervised classification method orthogonal partial least squares discriminant analysis (OPLS-DA) was utilized. This test showed how two variables differed and allowed enhanced interpretation of the metabolic variations between the pre- and post-treatment groups by removing information that had no impact on the discrimination and features. A variable importance in the projection (VIP) value of >1 was considered significant. Thereafter, Mass Profiler Professional software was used for univariate analysis. A volcano plot was used to identify the metabolites that significantly differed between the pre- and post-treatment groups, based on a *P*-value of <0.05 and a fold change (FC) cutoff of 2. Significantly altered features were chosen based on the univariate and multivariate analyses.

Next, the metabolites were putatively annotated based on the exact mass, isotopic distribution, and fragmentation pattern in different databases, including the Human Metabolome Database (https://hmdb.ca/) and METLIN (http://metlin.scripps.edu), within a mass difference of 5 ppm. Exogenous metabolites, such as those from drugs and environmental exposures, were excluded, and the remaining identified metabolites were retained for further pathway analyses. The receiver operating characteristic (ROC) curve was used to assess the diagnostic ability of the potential biomarkers to discriminate between a hypothyroid and a euthyroid state during LT_4_ treatment. Sensitivity, specificity, and area under the ROC curve (AUC) were determined using the MetaboAnalyst program (https://www.metaboanalyst.ca/).

## Results

### Biochemical analysis

The measurements at baseline and after treatment in a euthyroid state are listed in Table 1.

### Mass ion detection and significantly altered metabolites

A total of 20,406 mass ion features were detected (13,010 and 7396 in positive and negative 18 modes, respectively) (Supplementary Table 1, see section on supplementary materials given at the end of this article). After applying a frequency threshold (cutoff, 80% of all samples) and excluding 19% of missing values during the peak detection and alignment steps, 16,565 features were retained for statistical analysis. Afterward, these features were normalized by total signal median and then log-transformed and Pareto-scaled before univariate and multivariate analyses.

**Figure 1:**
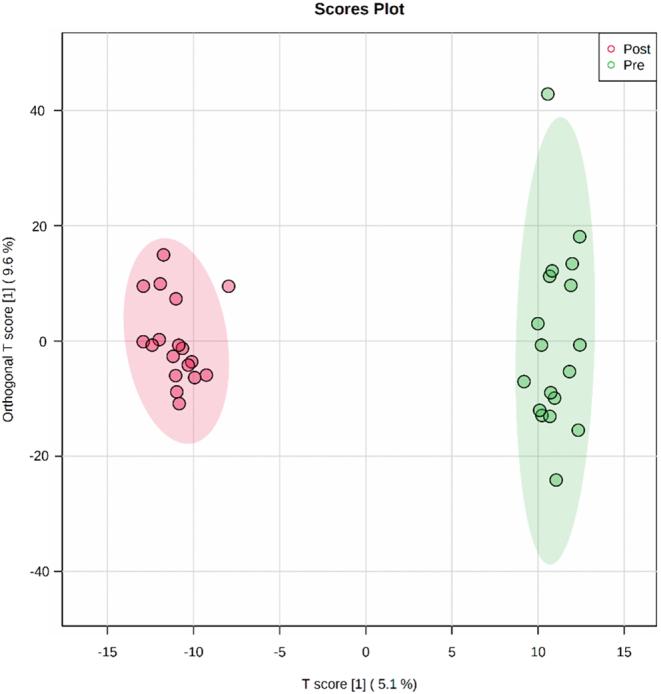
The orthogonal partial least squares discriminant analysis (OPLS-DA) model. There is evident group separation between the post- and pretreatment groups. The robustness of the created models is evaluated by the fitness of the model (R2Y = 0.991) and the predictive ability (Q2 = 0.404) values on larger data (*n* = 100). (green dots represent the pretreatment group, and red dots represent the post-treatment group).

Based on the multivariate analysis, the OPLS-DA model (Fig. 1) clearly showed separation between the two groups; this result highlighted that the metabolic profile of patients with hypothyroidism significantly differed before and after treatment. Using an extended dataset (*n* = 100), a permutation test to validate the OPLS-DA model showed R2Y = 0.991 and Q2 = 0.404. The discriminant mass ions with a VIP value of >1 were responsible for the proposed separation and were subjected to univariate analysis to determine their statistical significance.

**Figure 2:**
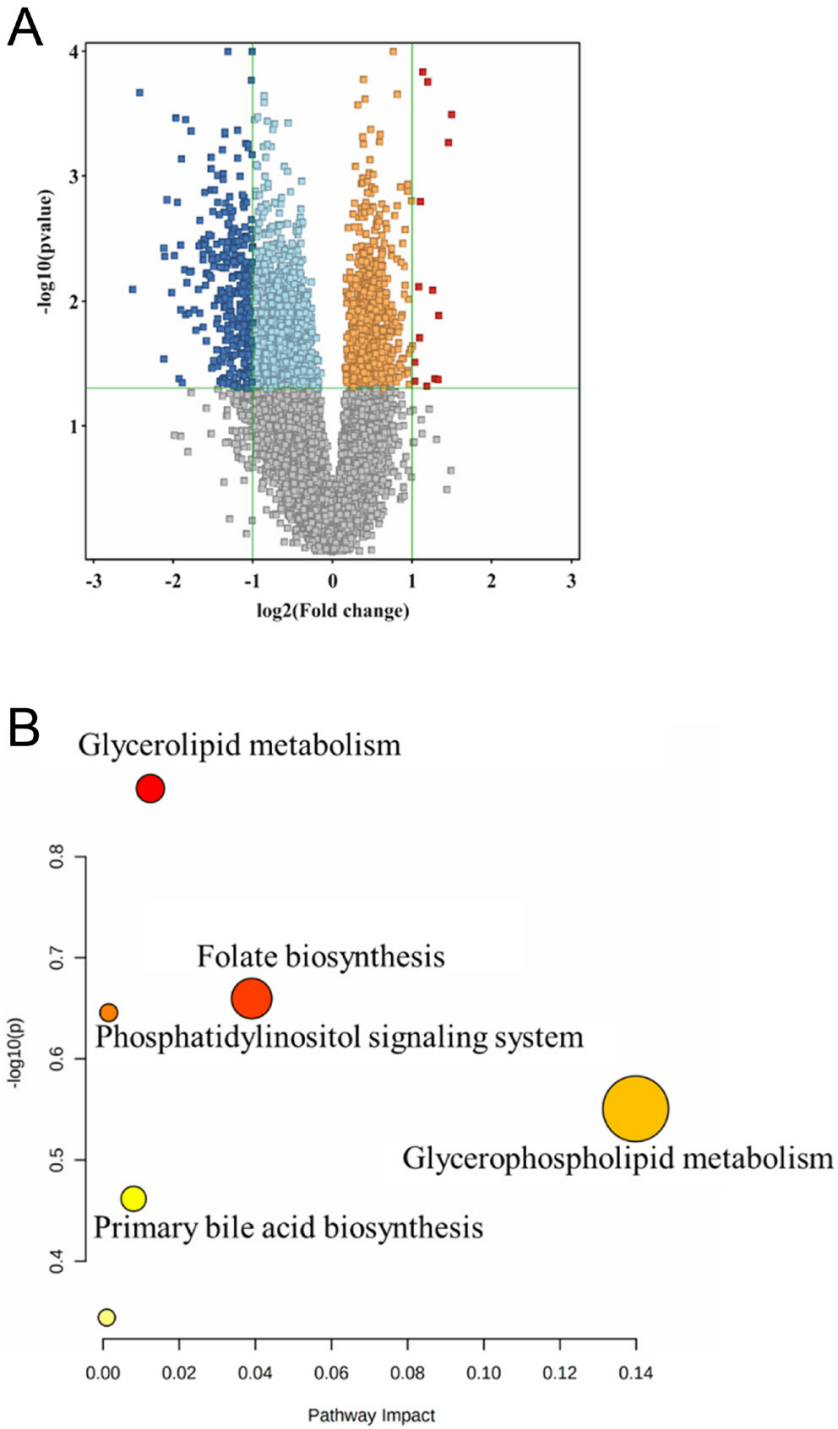
(A) This is a volcano plot between the post- and pretreatment groups after applying an 80% filter on all data, (paired *t*-test, no correction, *P* ≤ 0.05, FC 2). Of 334 dysregulated metabolites, 14 (red) are upregulated and 320 (blue) are downregulated between the two groups, respectively. Orange and light blue squares refer to metabolites that failed to pass fold change cutoffs and were up- and downregulated, respectively. Gray square metabolites failed to pass both cutoffs. (B) Pathway analysis of the significantly dysregulated metabolites of patients with hypothyroidism before and after treatment. Colors (varying from yellow to red) mean the metabolites are in the data with different significance levels (*P*-value).

On univariate analysis and volcano plot, 334 features were significantly dysregulated by LT_4_ treatment (Fig. 2A). Of these features, 14 were upregulated and 320 were downregulated after treatment (Supplementary Table 2). Supplementary Table 3 shows a summary of 210 of the 334 compounds identified. The top 94 metabolites were identified as endogenous and retained for further pathway and biomarkers analyses (Supplementary Table 4).

The folate biosynthesis; glycerolipid, glycerophospholipid, and phosphatidylinositol signaling systems; and primary bile acid biosynthesis were the most affected pathways between the two groups (Fig. 2B). Compared with the pretreatment group, the post-treatment group had a metabolic plasma profile of significantly higher levels of glycocholic acid, lysophosphatidylinositol (LysoPI) (16:0/0:0), monoacylglyceride (MG) (0:0/24:1(15Z)/0:0), 1,2-diglyceride (DG) (8:0/13:0/0:0), and triacylglycerol (TG) (14:0/14:0/18:1(11Z)) and significantly lower levels of diacylglycerol (DG) (24:0/20:2), phosphatidylcholine (PC) (18:1(12Z)-O(9S,10R)/2:0), asparagine (Asn), aspartic acid (Asp), methionine (Met), tetracosatetraenoyl carnitine, 3-hydroxyhexanoylcarnitine, 2-octanoic, and DG (16:1n7/0:0/18:2n6). These metabolites might be considered promising biomarkers for transitioning from hypothyroid to euthyroid following LT_4_ treatment.

**Figure 3:**
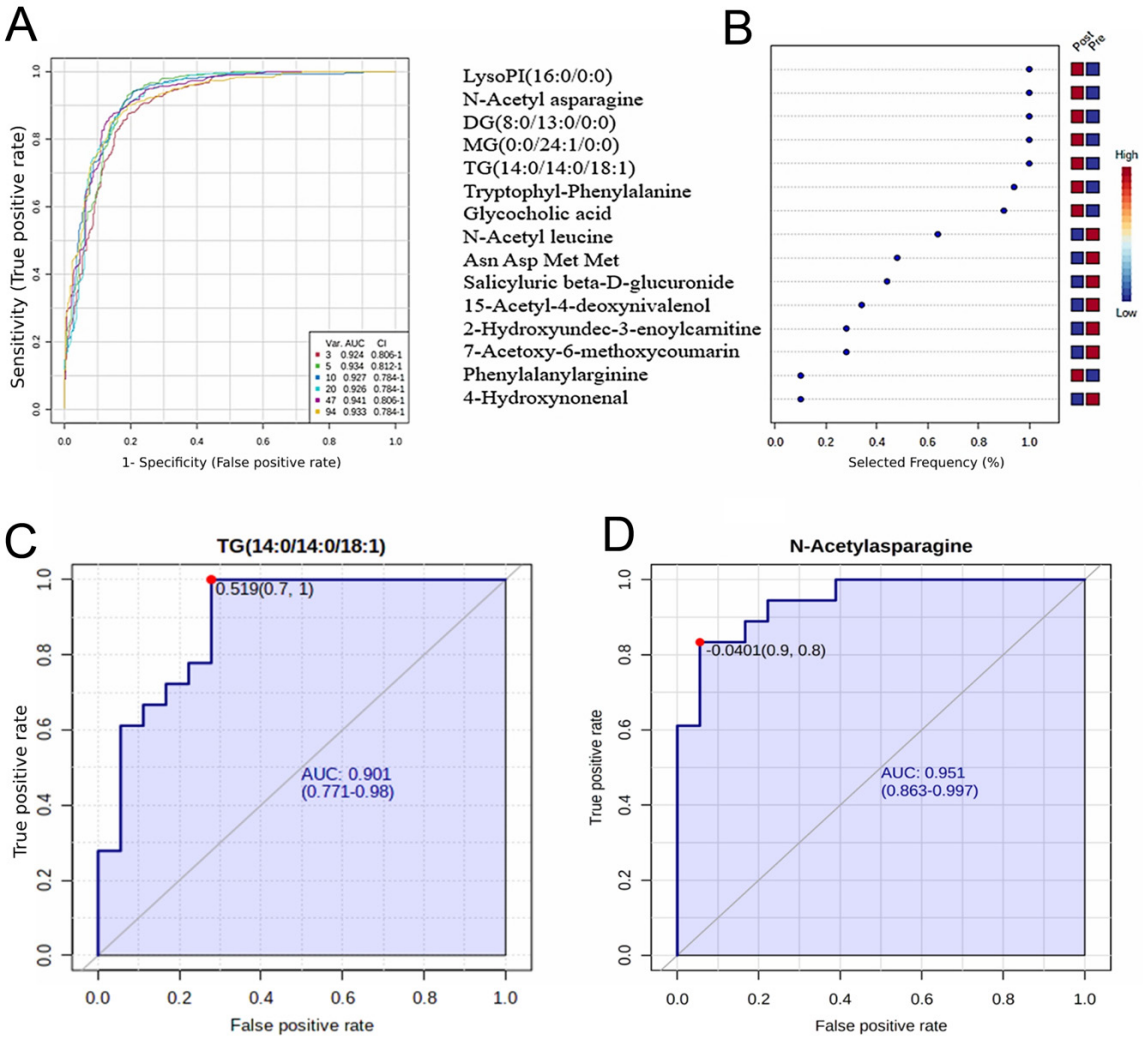
Receiver operating characteristics (ROC) curve was generated by the orthogonal partial least squares discriminant analysis (OPLS-DA) model, with area under the curve (AUC) values calculated from the combination of 5, 10, 15, 25, 50, and 100 metabolites and (B) loading for significantly altered metabolites before and after hypothyroidism treatment are shown. (C) TG(14:0/14:0l18:1) (AUC, 0.901) and (D) *N*-acetyl asparagine (AUC, 0.951) as examples of upregulated after hypothyroidism treatment.

Figure 3(A) shows the specificity and sensitivity of the potential biomarkers. Three features in the ROC curve had an AUC value of 0.924. Feature ranking in OPLS-DA (Fig. 3B) revealed that LysoPI(16:0/0:0), MG(0:0/24:1(15Z)/0:0), DG(8:0/13:0/0:0), TG(14:0/14:0/18:1(11Z)), and *N*-acetylasparagine were the most discriminative metabolites.

## Discussion

Thyroid hormones affect nearly every cell in the body when they cross the plasma membrane and bind to receptors on the mitochondria that control metabolism, including energy production and glucose oxidation (20, 21). In 1930, hypothyroidism was first reported to have a huge impact on blood lipid components (22). Since then, several studies that examined the association and impact of thyroid hormones on metabolism have shed light on the pathophysiology of hypothyroidism and the mechanism of action of LT_4_.

In this study, we demonstrated distinct sample clustering and group separation between the hypothyroid and euthyroid groups as a result of LT_4_ treatment, as shown in the OPLS-DA model, which had acceptable goodness of fit and prediction (23). Moreover, LysoPI(16:0/0:0), MG(0:0/24:1(15Z)/0:0), DG(8:0/13:0/0:0), and TG(14:0/14:0/18:1(11Z)) were the highest ranking metabolites that could discriminate between hypothyroidism and euthyroidism before and after treatment, respectively. Therefore, these metabolites may be further evaluated to monitor treatment with LT_4_. The significant increase in the T_4_ level, which is expected to significantly decrease the TSH level, in the euthyroid state from the T_4_ level in the hypothyroid state reflected a positive therapeutic effect of LT_4_ on hypothyroidism.

Inadequate levels of circulating thyroid hormones secondary to hypothyroidism decrease hepatic cholesterol synthesis, probably by inhibition of hydroxy methylglutaryl coenzyme (HMG-CoA) reductase. Furthermore, thyroid hormones are known to stimulate transcription of the LDL receptor and HMG-CoA reductase genes. HMG-CoA reductase is expressed in the liver and stimulates the production of cholesterol. However, a decrease in hepatic cholesterol synthesis is outweighed by the action of thyroid hormones on LDL cholesterol receptor expression, resulting in net accumulation of serum LDL cholesterol. Therefore, compared with healthy subjects, patients with hypothyroidism usually present with increased TC and LDL concentrations. Moreover, they manifest elevated TG levels secondary to a decrease in the activity of lipoprotein lipase, which is an enzyme that degrades circulating TG in the bloodstream (24, 25, 26, 27). LT_4_ can be extremely effective for the treatment of hyperlipidemia associated with hypothyroidism (26). However, our results showed no notable changes in the serum concentrations of TC, LDL, and TG after LT_4_ treatment. This implied that a longer time may be needed to observe for changes in the lipid profile, even if a euthyroid state has been achieved. Another reason for the absence of significant changes in the lipid profile after LT_4_ treatment may be the fact that the recruited patients had lipid profiles within the accepted range before treatment.

In addition to dyslipidemia, which is consistently seen in hypothyroidism, considerably greater plasma levels of glycerophospholipids in individuals with clinical and subclinical hypothyroidism than in euthyroid individuals were previously reported (9). Moreover, several animal studies reported that thyroid hormones could greatly affect sphingolipid metabolism, *de novo* ceramide synthesis, glycerophospholipid metabolism, and fatty acid β-oxidation in the liver (28, 29). In other studies, the level of ceramides increased upon LT_4_ administration to hypothyroid tissue and was even higher in hyperthyroid hepatocytes, liver tissue, and heart muscle (29, 30). LT_4_ is believed to increase the activity of ceramide phosphocholine transferase, which is an enzyme that transfers the phosphocholine group from phosphatidyl cholines (PC) to ceramide to generate sphingomyelin (SM) and diglycerides (DG) (31). In a recent plasma metabolomics profiling study by Feifei Shao *et al.*, the level of ceramide was higher in individuals with clinical and subclinical hypothyroidism than in the control group (9). Consistent with previous reports, this present study found low plasma levels of ceramides, trihexosyl ceramide (d18:1/22:0), and trihexosyl ceramide (d18:1/24:0) upon LT_4_ treatment. In addition, the downregulation of ten metabolites identified as PC following LT_4_ treatment could reflect that more PCs were redirected to the liver and consumed during SM synthesis, while the liver lipoprotein-associated PC efflux decreased. This provided evidence of the key role of thyroid hormones in lipid synthesis and metabolism, particularly sphingolipid metabolism in the liver. Moreover, monitoring plasma and/or liver ceramides and probably SM could serve as gold standard regulators of hypothyroid treatment and may be used to monitor LT_4_ overdose.

An elevated level of DG is a common finding in hypothyroidism. DG accumulation had been attributed to multiple routes, including lipid efflux, ceramide, polyphosphoinositide, and PC hydrolysis, in addition to reduced hepatic lipogenesis, all of which resulted in reduced levels of PC and TG (32). Sustained elevation of DG level is believed to be associated with decreased protein kinase C (PKC) activity (33). PKC comprises a family of closely related enzymes that are activated by the second messenger calcium (Ca^2+^) and DG, depending on the duration and magnitude of these signals. Inappropriate DG buildup promotes different cellular transformations, including hepatocarcinogenesis, hyperglycemia, hypertriglyceridemia, other cardiovascular complications of diabetes, and hypothyroidism (34, 35, 36, 37, 38). In addition, the work of Bansode R *et al.* on mouse liver and skeletal muscle showed reductions in adipose tissue depots and TG content upon deletion of the PKCβ isoform (37, 38). Therefore, the level of intracellular DG must be closely regulated. Upon LT_4_ administration, PKC is reactivated, the amount of DG synthesized de novo will be decreased, and the level of TG is expected to increase (33, 39, 40). In the current study, the levels of three of four identified DGs were downregulated and the level of one of two identified TGs was upregulated after treatment. Although this result coincides with that of previous studies, not all DGs and TGs follow the same trend, thereby, suggesting that a complete metabolic change was not achieved after reaching a euthyroid state with treatment, because T_3_ and T_4_ levels do not reflect all the changes reversed in hypothyroidism. Consistent with our findings, the results of C. Piras *et al.* showed that upon LT_4_ administration to 18 patients with overt primary hypothyroidism, the metabolic changes persisted despite the normalization of serum TSH and thyroid hormone concentrations (17). Therefore, the metabolomics technique may help integrate traditional hormone assays and more effectively assess the accomplishment of a euthyroid state.

Thyroid hormones are known to increase the transcription of fibroblast growth factor 21 (FGF21), which is a coded protein that serves as an endocrine factor and a key metabolic regulator of glucose and lipids in adipose tissue (41). A reduced level of FGF21 was reported in hypothyroidism, whereas an elevated FGF21 level was observed in hyperthyroidism (42, 43). FGF21 inhibits the synthesis of fatty acids, cholesterol, and TG and stimulates fatty acid uptake and β-oxidation, which enhance thermogenesis and increase energy expenditure (44, 45). β-oxidation is a multistep process that takes place in the mitochondria, where fatty acids are broken down by different tissues to produce acetyl-CoA units that can be employed in the tricarboxylic acid (TCA) cycle to produce ATP. Therefore, accumulation of plasma long-chain acylcarnitine in patients with hypothyroidism supports the decrease in the activity of fatty acid oxidation, which may indicate dysfunction in the long-chain fatty acid transport protein (FATP) 1 (28) or incomplete β-oxidation relative to the TCA cycle flux (46). In this study, a low level of long-chain acylcarnitine was detected upon treatment; this reflected enhanced β-oxidation in euthyroid patients than in hypothyroid patients. Thyroid hormones activate Ca^2+^/calmodulin-dependent protein kinase-β, which, in turn, mediates phosphorylation of AMP-activated protein kinase (AMPK) and increases fatty acid transport from the periphery into the cells by translocation of CD36 through activation of Ras-related protein Rab-8A** (**Rab8a) or FATP on the plasma membrane. In addition, AMPK activates acetyl-CoA carboxylase (ACC), which is an enzyme that catalyzes the conversion of acetyl-CoA to malonyl-CoA, which promotes fatty acid synthesis (47). However, when activated by AMPK, ACC activity is inhibited, thereby decreasing the amount of malonyl-CoA released and activating carnitine palmitoyl transferase 1, which is an outer mitochondrial membrane protein that converts acyl-CoA to acyl carnitine, accompanied by an increase in fatty acid trafficking into mitochondria (47). In addition, thyroid hormones increase the production of several mitochondrial enzymes required for β-oxidation, including mitochondrial uncoupling protein (48), pyruvate dehydrogenase kinase (49), and medium-chain acyl-CoA dehydrogenase (50).

Furthermore, glycocholic acid was increased upon restoration of a euthyroid state. The thyroid hormone facilitates the conversion of cholesterol to bile acid by stimulating the rate-limiting enzyme cholesterol 7α-hydroxylase through hepatic TH β receptors and stimulates bile acid secretion in the liver and intestine (51, 52, 53). Therefore, an increase in the glycocholic acid concentration was expected in the post-treatment samples.

Compared with a hypothyroid state, a euthyroid state led to the downregulation of small peptides upon achieving normal plasma FT_4_; this suggested a change in protein metabolism. Since 1956, whole-body protein breakdown and decreased synthesis had been documented upon disruption in thyroid hormone levels (either hypothyroidism or hyperthyroidism). However, the reversal of these changes following treatment indicated the importance of sustaining normal thyroid hormone levels for maintaining anabolic effects (54, 55).

The findings from the study have to be taken into consideration keeping in mind the small sample size and lack of gender base analysis and the time duration to reach euthyroid levels. Another aspect that requires consideration is the observation that our study demonstrates notable enhancements in the metabolomic profile following thyroxine treatment. However, it remains uncertain at which levels of FT4 there is a full normalization of the metabolomic profile, representing genuine cellular euthyroidism, rather than simply falling within the laboratory's normal FT4 range. Additional research is imperative to validate the findings obtained in this study.

## Conclusions

A metabolomic analysis of patients with hypothyroidism revealed significant changes in the level of several metabolites and lipids after treatment. This study showed the value of the metabolomics technique in providing a complementary understanding of the pathophysiology of hypothyroidism and as a crucial tool for examining the molecular impact of LT_4_ treatment on hypothyroidism. However, it is important to note that metabolic changes might persist upon reaching a euthyroid state after treatment.

## Supplementary Materials

Supplementary Tables

## Declaration of interest

The authors declare that the research was conducted in the absence of any commercial or financial relationships that could be considered as potential conflict of interest.

## Funding

This project was funded by the National Plan for Science, Technology and Innovation http://dx.doi.org/10.13039/501100005725 (MAARIFAH), King Abdulaziz City for Science and Technology http://dx.doi.org/10.13039/501100004919, Saudi Arabia (Project No. 08-MED513-02).

## Data availability statement

Metabolomics data were deposited to the EMBL-EBI MetaboLights database with the identifier MTBLS7600. The complete dataset can be accessed at https://www.ebi.ac.uk/metabolights/MTBLS7600 (30 March 2023).

## Statement of ethics and informed consent statement

The study was conducted in accordance with the Declaration of Helsinki, and all procedures and protocols, including the clinical samples, were reviewed and approved by the ethics committee of the College of Medicine, King Saud University, Riyadh, Saudi Arabia (registration no. E-10-172). Informed consent was obtained from all participants involved in the study.

## Author contribution statement

Hicham Benabdelkamel, Anas M. Abdel Rahman, and Assim A. Alfadda conceived the idea and designed the study. Assim A. Alfadda and Afshan Masood were involved in patient recruitment. Hicham Benabdelkamel, Anas M. Abdel Rahman, Reem H. AlMalki, and Mohthash Musambil performed the metabolomics laboratory work. Anas M. Abdel Rahman, Reem H. AlMalki, and Lina A. Dahabiyeh carried out data analysis. Hicham Benabdelkamel, Malak A. Jaber, Lina A. Dahabiyeh, Afshan Masood, Reem H. AlMalki, Anas M. Abdel Rahman, and Assim A. Alfadda wrote the manuscript. All authors have read and approved the final manuscript.
